# Kahramanmaraş-Pazarcık Earthquake 2023: Characteristics of Patients Presented to the Emergency Department of a Tertiary Hospital Far from the Region and Infection Characteristics in Hospitalized Patients

**DOI:** 10.1017/S1049023X24000062

**Published:** 2024-02

**Authors:** Özlem Çakın, Melike Yüce Aktepe, Samet Acar, Süleyman İbze

**Affiliations:** 1. Akdeniz University, Faculty of Medicine, Department of Internal Medicine Intensive Care Unit, Antalya, Türkiye; 2. Akdeniz University, Faculty of Medicine, Department of Infectious Diseases and Clinical Microbiology, Antalya, Türkiye; 3. Akdeniz University, Faculty of Medicine, Department of Emergency Medicine, Antalya, Türkiye

**Keywords:** earthquake, emergency department, infection, patient management

## Abstract

**Objective:**

The aim of this study is to determine the demographic, clinical characteristics, and outcomes of the patients who applied to the emergency department (ED) of Akdeniz University Faculty of Medicine Hospital (Antalya, Türkiye) after the Kahramanmaraş-Pazarcık earthquake dated February 6, 2023, as earthquake victims were included in the study. The results of the study could be a guide in terms of emergency health services and the healthy management of disasters.

**Methods:**

The study included patients over the age of 18 who presented as earthquake victims to the ED of Akdeniz University Medical Faculty Hospital from February 6, 2023 through March 8, 2023. The demographic data of the patients, including age, gender, earthquake zone, time and manner of arrival to the ED, time under debris, length-of-stay (LOS) in the service and intensive care unit (ICU), infection rates, culture results, and mortality, were retrospectively analyzed using the hospital automation system.

**Results:**

A total of 1,833 earthquake victims presented to the ED. Of these patients, 1,294 were adults and 539 were children. Services and the ICU admitted a total of 137 adult patients. In the first week, 414 (31.99%) of the patients presented to the ED, while 82 (59.85%) of the hospitalized patients were admitted.

Hatay ranked first with 573 (44.28%) patients in the distribution of patients presented to the ED according to earthquake regions. In the distribution of hospitalized patients by earthquake regions, the patients requiring the most hospitalization were from the province of Hatay, with 68 (49.63%) patients.

During hospital observations, the medical staff took 132 culture samples based on the positive clinic of the patient. The microorganisms detected in the culture studies were different from the flora of the hospital. The mortality at seven days was two (1.45%), and at the end of 30 days, the mortality was six (4.37%).

**Conclusions:**

The ED evaluated all affected cases, with most patients being brought by their relatives using their own means, and had low mortality rates despite presenting with fewer injuries. New environmental conditions that developed after the earthquake caused unexpected results, especially in terms of community-acquired agents.

## Introduction

Major earthquakes are one of the most destructive natural disasters. The epidemiology of injuries and deaths is unique among disasters. Earthquakes affect crowded urban areas with poor structural standards, creating mass effects that cause many traumatic injuries and deaths. Mechanical effects cause multiple traumas resulting in these injuries. Patients require intensive curative medical and surgical care at a time when their local and regional capacity for medical intervention is at least partially impaired. Many patients who survive blunt and penetrating trauma and crush injuries have subsequent complications leading to additional morbidity and mortality.^1^ Türkiye is on the Mediterranean-Alpine-Himalayan seismic belt. Ninety-three percent of Türkiye’s land is in the earthquake zone, and 98% of the population lives in areas under threat from earthquakes.^2^ The Kahramanmaraş-Pazarcık earthquake, which occurred on February 6, 2023 at 4:17am Türkiye time with coordinates latitude: 37,288, longitude: 37,043, and depth: 8,60 km and a magnitude of 7.7 Mw on the Richter scale, went down in history as one of the biggest disasters of the last century. After the first earthquake, approximately nine hours later, at 1:24pm Türkiye time, the second earthquake with a magnitude of 7.6 Mw, which occurred in Elbistan-Kahramanmaraş-Türkiye, shook the region again, followed by a third earthquake with a magnitude of 6.4 Mw. Between the main shock and February 9, 2023 at 4:00pm, seismologists recorded nearly 1,300 earthquakes.^3^ By June 19, 2023, authorities had announced a total of 50,500 casualties.^4^ Health workers were also seriously injured in this devastating disaster, and according to the statement made by the Minister of Health, a total of 448 health workers died in the earthquake. According to investigations by the Turkish Medical Association (Ankara, Türkiye) and Sağlık ve Sosyal Hizmet Emekçileri Sendikası (SES [Health and Social Service Workers Union]; Ankara, Türkiye), at least 101 of those who died were physicians.^5^

The main cities affected by the earthquake were Kahramanmaraş, Hatay, Malatya, Gaziantep, Adana, Osmaniye, Diyarbakır, Şanlıurfa, Adıyaman, and Kilis. The earthquake destroyed or severely damaged over 100,000 buildings in the affected area.

After the earthquake, the surrounding provinces treated a large number of earthquake victims due to the severe damage to the region and its health system. Antalya became one of the cities where earthquake victims applied. Antalya is 795km from Kahramanmaraş, the epicenter of the earthquake, and 792km from the city center of Hatay, where the destruction was most severe. The study aimed to determine the general characteristics and infection profiles of patients who presented to the emergency department (ED) of a tertiary health care institution located far from the earthquake area and subsequently required hospitalization in the service and intensive care unit (ICU).

## Methods

This study was carried out as a single-center, cross-sectional retrospective study. The study included patients who were 18 years of age and older and sought treatment at the ED of Akdeniz University Faculty of Medicine Hospital (Antalya, Türkiye) within one month after the earthquake. Patients under the age of 18 were excluded from the study. This study was approved by the Akdeniz University Clinical Research Ethics Committee and obtained data usage permission (approval number: KAEK-235; dated March 22, 2023). Researchers maintained the confidentiality of patient data throughout the study and adhered to the ethical principles of clinical studies according to the Declaration of Helsinki. The data of adult patients who were presented to the ED with the earthquake victim diagnosis code (ICD code: X34) were recorded. The information management system “MiaMed” (MIA Teknoloji A.Ş.; Ankara, Türkiye) used in the hospital recorded and monitored the service and/or ICU follow-up records of the patients. The demographic data of the patients, including age, gender, earthquake region, time of arrival to the ED and type of arrival, duration of the wreck, hospitalization status, length-of-stay (LOS) in the services and ICU, infection rates, culture results, and mortality, were evaluated.

The Infection Control Committee evaluated the records of hospitalized patients daily. Patients were classified as those who developed infection in the first 48 hours of hospitalization, those who developed infection after 48 hours, and those who did not develop infection. Mortality rates of the patients were recorded the in the first seven days and 28 days following their hospitalization, and the factors affecting mortality by dividing the patients into two groups, living and dead, were evaluated.

The data were analyzed using IBM SPSS Statistics 25.0 (IBM Corp.; 2017 IBM SPSS Statistics for Windows, Version 25.0; Armonk, New York USA). Numerical variables were presented as mean and standard deviation (SD) or median (minimum, maximum), and categorical variables were presented as number (n) and percentage (%). Normal distribution of numerical variables was examined using the Shapiro-Wilk test. Numerical variables were analyzed using Student’s T test or Man-Whitney-U test. Pearson Chi-square or Fisher exact test was used for analysis of categorical variables. The significance level was accepted as 0.05 for all hypotheses.

## Results

**Figure 1.**
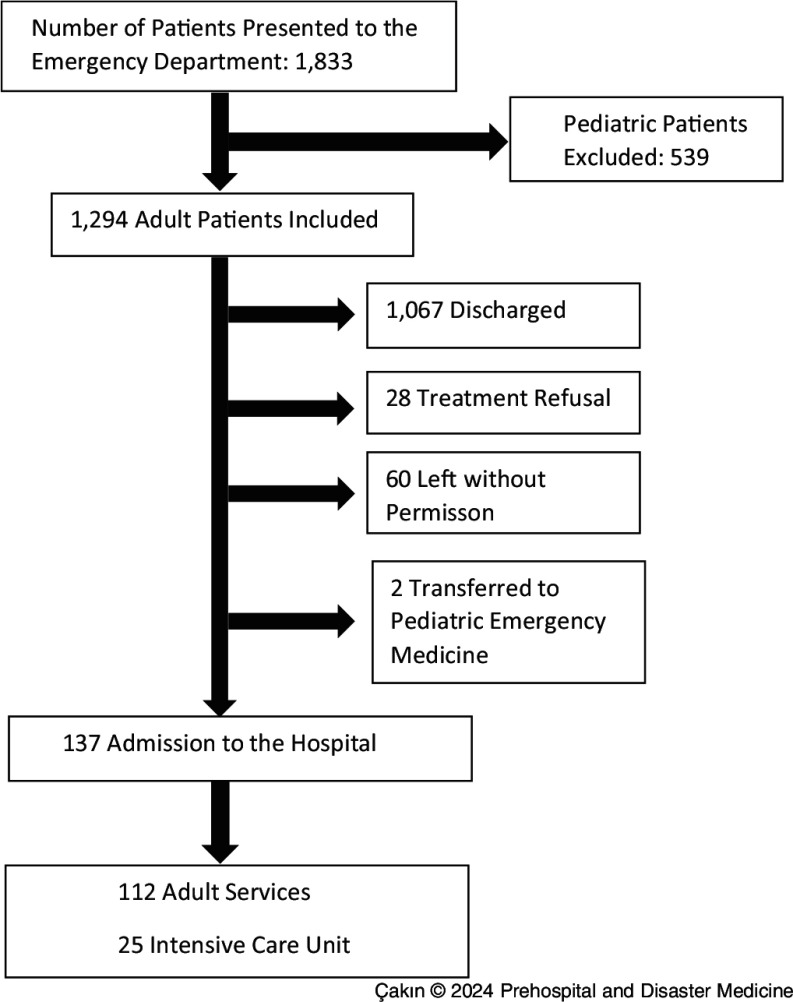
Study Flow Chart.

Within one month after the earthquake, 1,833 patients were presented to the ED of Akdeniz University Faculty of Medicine Hospital. While 1,294 of these patients were adults, 539 of them were children under the age of 18. A total of 137 patients were admitted to the service and ICU. Figure 1 shows the ED admissions and outcomes of the patients included in the study.

**Figure 2.**
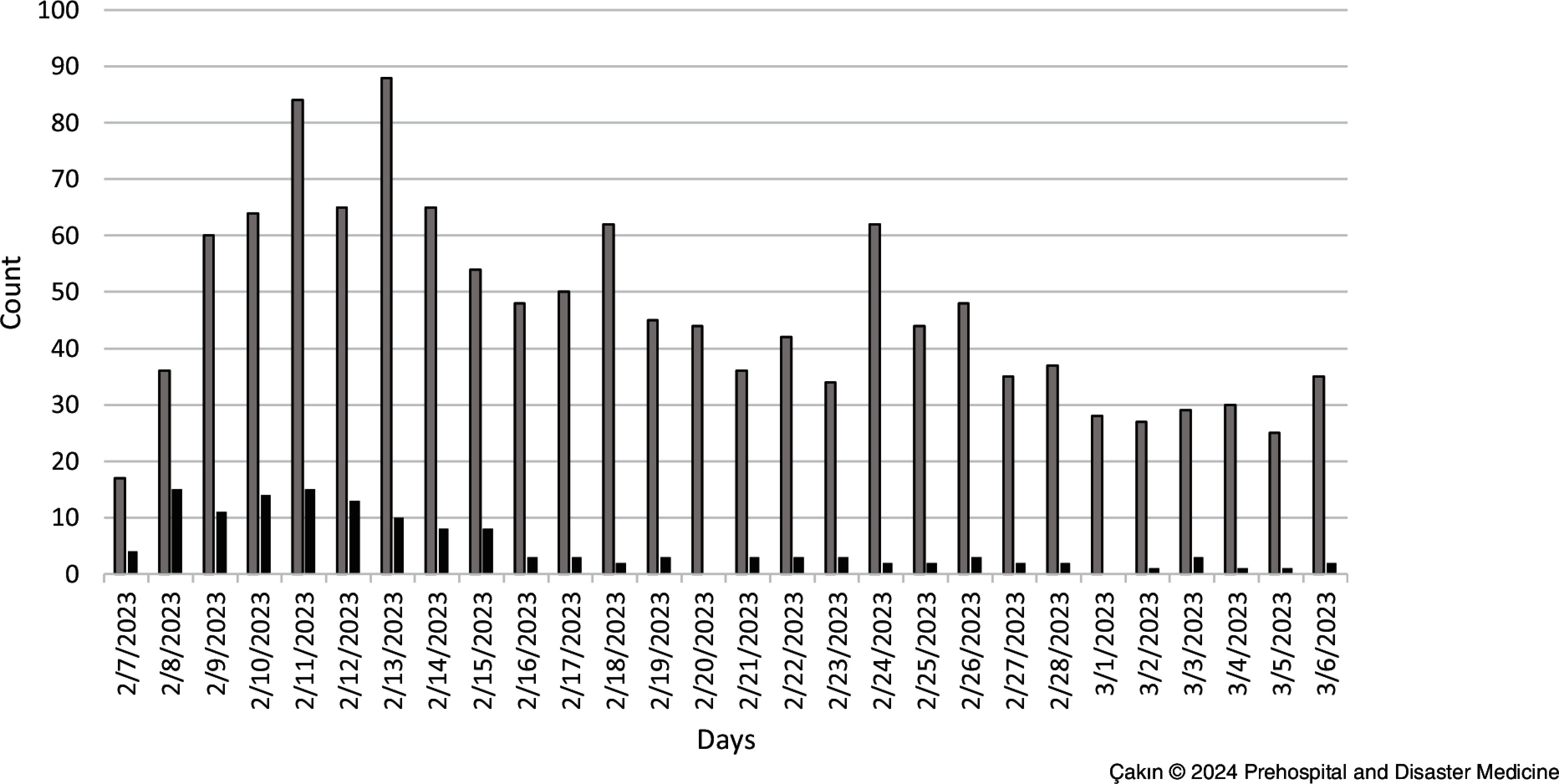
Distribution of Patients Presented to the Emergency Department and Hospitalized Patients by Days. Note: Gray columns show the number of presented patients, black columns show the number of inpatients.

Of the patients who presented to the ED in the first week, 414 (31.99%) were admitted, and 82 (59.85%) of the hospitalized patients were admitted within the same week, with a subsequent decrease in the admission rate. Figure 2 shows the distribution of patients who presented to the ED and were hospitalized by days.

Of the 1,294 patients who presented to the ED, 701 (54.17%) were women, and the mean age was 53.11 (SD = 19.03) years. The highest application was from Hatay with 573 (44.28%) patients, Kahramanmaraş with 260 (20.09%), and Malatya with 122 (9.40%) patients. One patient’s data could not be accessed due to unauthorized abandonment. Of the patients presented to the ED, 108 (8.34%) were presented by ambulance, while this figure was 19 (13.86%) in the patients admitted to the service.

A total of 137 patients were hospitalized in adult services and the ICU. Seventy-two (52.55%) of these patients were women, and the mean age of all patients was 44.05 (SD = 18.81) years. The highest number of patient admissions was Hatay with 68 (49.63%), followed by Kahramanmaraş with 22 (16.05%) patients and Adıyaman with 17 (12.40%). There were no applications from the four provinces affected by the earthquake. The orthopedic clinic had the most admissions, with 38 (27.73%) patients, while 20 (14.59%) of these patients were admitted to the ICU. Table 1 displays the demographic characteristics of the patients.

**Table 1.** Demographic Characteristics of Patients Presented to the Emergency Department and Subsequently Hospitalized

	Patients Presented to the Emergency Department	Patients Admitted to Hospital
Age	53.11 (SD = 19.03)	44.05 (SD = 18.81)
Gender		
Female	701 (54.17%)	72 (52.55%)
Male	593 (45.82%)	65 (47.44%)
Total	1294 (100.00%)	137 (100.00%)
Earthquake Region		
Hatay	573 (44.28%)	68 (49.63%)
Kahramanmaraş	260 (20.09%)	22 (16.05%)
Malatya	122 (9.40%)	9 (6.56%)
Adıyaman	118 (9.11%)	17 (12.40%)
Gaziantep	112 (8.65%)	18 (13.13%)
Adana	35 (2.7%)	1 (0.72%)
Diyarbakır	26 (2.00%)	2 (1.45%)
Şanlıurfa	21 (1.62%)	0 (0.0%)
Osmaniye	19 (1.46%)	0 (0.0%)
Kilis	4 (0.30%)	0 (0.0%)
Elâzığ	3 (0.23%)	0 (0.0%)
Unknown	1 (0.07%)	0 (0.0%)
Total	1294 (100.00%)	137 (100.00%)
How to Apply		
Ambulatory	1186 (91.65%)	118 (86.16%)
By Ambulance	108 (8.34%)	19 (13.86%)
Total	1294 (100.00%)	137 (100.00%)
Admitted Unit		
Intensive Care		20 (14.59%)
Orthopedics		38 (27.73%)
Internal Medicine		25 (18.24%)
Pulmonary Diseases		16 (11.67%)
Neurosurgery		10 (7.29%)
Obstetrics & Gynecology		10 (7.29%)
Other Units		18 (13.13%)

Note: Data are expressed as number of people and percentage (%). Age expressed as mean and standard deviation.

During the hospital observations, the medical team took 132 culture samples based on the positive clinical status of the patient. Those evaluated as positive results from the samples taken within the first 48 hours were classified as community origin and external center origin. Cultures taken 48 hours after hospitalization and whose incubation period had not started before were considered to be of nosocomial origin. There were 34 cultures of nosocomial origin.

All culture development was examined and a total of 11 tracheal cultures and smears were taken. Six of them showed significant growth. Of those with growth, four were from intubated patients (Klebsiella, Acinetobacter, and Moraxella) and two were from patients who were not intubated but who needed frequent aspiration (Methicillin-resistant Staphylococcus aureus [MRSA] and Pseudomonas).

Pus and wound cultures were taken nine times. Reproduction was detected in seven of them. Three of these growths were detected post-operatively (Acinetobacter, Pseudomonas, and Stenotrophomonas), one of them intra-operatively (Enterobacter cloacae), and three of them in pre-operative samples taken secondary to wound, crush, or trauma (Pseudomonas, Acinetobacter, and Staphylococcus aureus).

Table 2 presents the positive culture results obtained within 48 hours after hospitalization and during the first 48 hours. The LOS in the ICU of patients with nosocomial infection was 6.37 (SD = 9.00) days (P = .000), and the 30-day mortality rate was three (18.8%) people (P = .002) and significantly higher. Table 3 presents the data.

**Table 2.** Results of Culture Samples

	Culture Samples Studied within the First 48 Hours	Culture Samples Studied 48 Hours After Hospitalization
Urine Culture	Pseudomonas aeruginosa Enterocuccus faecıum (2) Escherichia coli	Candida albicans (3) Candida parapsilosis Candida tropicalis Escherichia coli (4) Enterocuccus faecıum (3) Klebsiella species (2) Acinetobacter baumannii Chryseobacterium indologenes Citrobacter freundii
Blood Culture	Proteus species MRSA	Klebsiella pneumoniae (3) CNS Candida glabrata
Catheter Culture		Klebsiella pneumoniae Stenotrophomonas maltophilia
Sputum Culture	Haemophilus influenzae Candida albicans	Pseudomonas aeruginosa Candida tropicalis
Tracheal Culture	Moraxella catarrhalis MRSA	Klebsiella pneumoniae (2) Acinetobacter baumannii Pseudomonas aeruginosa
Wound Pu Culture	Stenotrophomonas maltophilia Staphylococcus aureus Pseudomonas aeruginosa	Enterobacter cloacea Acinetobacter baumanii (2) Pseudomonas aeruginosa

Abbreviations: CNS, Coagulase negative staphylococcus; MRSA, Methicillin-resistant staphylococcus aureus.

**Table 3.** Factors that Increase the Risk of Infection in Hospitalized Patients and their Effect on Mortality

	Infection during Hospitalization (n = 16)	No Infection during Hospitalization (n = 112)	P Value
Age, year	50.06 (SD = 22.54)	52.17 (SD = 18.68)	.151
Gender, n (%)			.461
Female	10 (62.5%)	59 (52.7%)	
Male	6 (37.5%)	53 (47.3%)	
Time Under Debris, minute	547.81 (SD = 1081.35)	281.38 (SD = 1010.16)	.154
Service Hospitalization, day	20.31 (SD = 22.02)	10.68 (SD = 10.22)	.468
Intensive Care Hospitalization, day	6.37 (SD = 9.00)	0.41 (SD = 1.27)	0
30-Day Mortality	3 (18.8%)	0 (0.00%)	.002

Note: Age is expressed as mean and standard deviation. Gender was expressed as number of persons and percentage (%). P <.05 is significant.

While the mortality rate was two (1.45%) people in the first seven days, the mortality rate was six (4.37%) people at the end of 30 days. The LOS in the ward, the LOS in the ICU, the age of the patient, and the presence of a nosocomial infection were effective in reducing mortality. Table 4 displays the data.

**Table 4.** Factors Affecting Mortality

	Alive (n = 131)	Dead (n = 6)	P Value
Age, year	52.86 (SD = 18.81)	71.00 (SD = 22.67)	.025
Gender, n (%)			
Female	70 (53.4%)	2 (33.3%)	.423
Male	61 (46.6%)	4 (66.7%)	
Time Under Debris, minute	285.49 (SD = 982.56)	480.0 (SD = 1175.75)	.489
Length-of-Stay in Ward, day	12.44 (SD = 12.49)	4.83 (SD = 9.94)	.007
Length-of-Stay in ICU, day	0.78 (SD = 2.90)	12.16 (SD = 7.13)	.000
Presence of Nosocomial infection	13 (9.9%)	3 (50.0%)	.021

Note: Age was expressed as mean and standard deviation, gender as number of persons and percentage (%). P <.05 is significant.

Abbreviation: ICU, intensive care unit.

## Discussion

The Kahramanmaraş earthquake is one of the most destructive earthquakes in history. Due to the damage to the highways, the accumulation of snow on the roads caused by the winter season, and the high density of civilian vehicles, it was not possible to reach the earthquake zone on the first day of the earthquake. In the early period, despite the development of various alternative routes, transportation remained unavailable. On February 12, 2023, the repair of the broken runway at Hatay Airport in the region was completed, and it opened to civilian flights. However, it did not cause any damage that would prevent flights at other airports. Military helicopters transferred medical personnel from Adana Incirlik Air Base to other disaster-affected regions. The transportation of health workers to disaster areas was the most frequently mentioned problem on the first day of the disaster. A total of 145km of road and tunnel damage in the region, damage to 1,204km of railway network, and the fire that broke out at the Iskenderun Port brought transportation and dispatch operations to a standstill.^6,7^ Out of all the patients who presented to the Akdeniz University ED, 1,669 (91.05%) arrived by their own means; 1,186 (91.65%) of the adult patients applied on their own. While 118 (86.13%) of the hospitalized patients arrived by their own means and private vehicles, 19 (18.86%) patients were transported by ambulance. In the study conducted by Eyler, et al after the October 30, 2022 Izmir earthquake, only 13% of the applications were made by ambulance, due to the low number of patient admissions to the emergency services, the relative distance of their hospitals to the region most affected by the earthquake, the difficulty of transportation due to the panic experienced after the earthquake, and the fact that there is a university hospital very close to the earthquake area.^8^

During the first three days, 113 (8.73%) of the patients presented to the ED were admitted, and within the first week, 414 (31.99%) were admitted. Within the first three days, 30 (21.89%) of the inpatients were completed, and by the end of the first week, 82 (59.85%) were completed. In the study by Sever, et al, 423 (70.1%) of the patients applied to reference hospitals in the first three days of the disaster. By the end of the first week, the hospitals admitted 562 patients, accounting for 93.2% of all patients.^9^ Seventy-five percent of the injured were hospitalized for the first three days after the 1995 Hanshin-Awaji earthquake, and the number of those who got sick continued to increase during the 15-day period after the earthquake.^10^ In this study, the rates were similar, and most of the applications were completed within the first seven days. When focusing on the provinces where earthquake victims came from, Hatay ranks first with 573 (44.28%) patients in all EDs, followed by Kahramanmaraş with 260 (20.09%) patients. Evaluation of hospitalized earthquake victims according to their province of origin, Hatay ranks first with 68 (49.63%) patients, followed by Kahramanmaraş with 22 (16.05%) patients and Gaziantep with 18 (13.11%) patients. The hospitalization of the patients after admission was orthopedics in the first place with 38 (27.73%) patients, internal medicine, especially nephrology with 25 (18.24%) patients, and intensive care with 20 (14.59%) patients.

There were eight university hospitals, 12.5% of the hospitals affiliated with the Ministry of Health, and 17.5% of the primary health care facilities within the earthquake zone. Within the earthquake zone, 16.5% of the total specialists and general practitioners serving in the hospitals affiliated with the Ministry of Health in Türkiye, as well as 15.5% of the other health employees, were working in the 11 affected provinces.^7^ The influx of injured in Malatya, Adıyaman, Kahramanmaraş, and Hatay, heavily affected by the earthquake, caused an insufficient number of health workers. The loss of lives, families, and homes prevented many health workers from actively working, leading to deficiencies in the first days. The lack of coordination in disaster areas and the arrival of the winter season delayed the arrival of health workers from outside the disaster areas.^6^

It is well-known that trauma patients have a high risk of developing infections and that in-hospital infection-related mortality increases in the presence of trauma. During the study period from August 17, 1999 through September 25, 1999, Öncel, et al observed that patients spent varying amounts of time under debris, ranging from six to 135 hours. Of the 143 culture samples taken, 48 were positive. Acinetobacter baumannii (31.2%) was in first place with 15 patients, followed by Staphylococcus aureus (18.7%) with nine growths and Pseudomonas aeruginosa (14.6%) with seven growths.^11^ Tao, et al conducted a study where 58.5% of the 775 cultured patients had positive results in pus and/or wound culture, with Gram-negative bacilli being the most frequently isolated pathogens.^12^ Forty-four of the patients admitted in this study had a history of being under debris. The longest time under debris was 145 hours and the shortest was 15 minutes; the average duration of the wreckage was 14.17 hours. The duration of the wreck was not risky for mortality or in-hospital infection development (P = .154; P = .489). In patients with in-hospital infection, the mortality rate was calculated as three (18.8%), which is consistent with the literature. Researchers detected microbial proliferation in 67 (60.0%) of the samples in the study conducted on patients with crush syndrome after the Marmara earthquake. Researchers isolated 67% Gram-negative bacilli, 17% Gram-positive cocci, 12% Enterobacteriaciae, and four percent yeast-like fungi from the wound samples. The main bacterial isolates for wound infections were Acinetobacter and Pseudomonas aeruginosa.^13^

Considering the flora of the hospital, the growths in the group that were considered community-acquired infections were incompatible with hospital flora, while the growths that were considered nosocomial infections were partially compatible. Recently, the most common agents encountered in this hospital flora are gram-negative bacilli: Klebsiella pneumoniae is in the first place (14%), Pseudomonas aeruginosa is in the second place (12%), and Acinetobacter baumanii is in the third place (10%). As seen in this study, the most common pathogenic microorganisms in hospitals were the main cause of infections thought to be nosocomial. This was especially true for repeated tracheal and wound pus cultures. Resistant gram-positive agents such as MRSA or Vancomycin-resistant Enterococcus (VRE) Faecium were rarely encountered in the hospital flora or in the growths in the study.

When the culture growths in the group considered as community-acquired infection were evaluated, some resistant microorganisms that were not expected to be encountered in community-acquired infections were encountered. Escherichia coli is the most common cause of community-acquired urinary tract infections detected in the urine culture taken in the first 48 hours. However, E. faecium and P. aeruginosa, which are the factors expected in nosocomial urinary tract infections, were encountered in the culture growths taken in the first 48 hours of the study. In the cultures taken after 48 hours, Candida albicans and non-Albican Candida come to the fore, especially due to long-term Foley catheter use, which is an expected situation. It has been revealed that Klebsiella species and Citrobacter fruendii, which produce Extended Spectrum Beta-Lactamase (ESBL) enzymes, which are frequently induced especially due to the empirical use of ceftriaxone, are resistant to third-generation cephalosporins compatible with hospital flora.

M. catharalis and H. influenza, which are the most common gram-positive and gram-negative agents in community-acquired pneumonia, were encountered in the samples taken from respiratory tract isolates. However, MRSA growth, which was expected to be the cause of nosocomial pneumonia, occurred in some patients. Although community-acquired MRSA is not very common, comorbidities such as diabetes, hemodialysis, and immunosuppression are also risk factors.^14^

When the patients in the study were examined, it was seen that the patient with MRSA growth was 80 years old and had diabetes and laryngeal cancer, which are among the risk factors for community-acquired MRSA. It was observed that the growths in the respiratory isolates taken 48 hours later were compatible with the hospital flora and had similar resistance rates.

Staphylococci and streptococci come to the fore in community-acquired wound infections. In this study, although it was initially thought that patients would have Staphylococcus infections, the presence of hospital-acquired wound infection agents such as S. malthophilia and P. aeruginosa was found to be greater than expected. Patients coming from the community with trauma such as penetrating or gunshot wounds, burns, crushing, or freezing may encounter gram-negative factors such as P. aeruginosa.^15^ Cultures taken after 48 hours are generally postoperative cultures and non-fermentative grams-negative bacilli are common and compatible with hospital flora.

Community-acquired MRSA was encountered when evaluating blood and catheter cultures in this group, but these factors are generally expected in both groups. Klebsiella pneumoniae, which is encountered in the hospital flora, especially in catheters that are not changed for more than seven days in the femoral region, is an expected factor and was the most common catheter-related infection agent in the study. The presence of Candida glabrata was detected in the cultures taken 48 hours after the patient, who had been catheterized for a long time, received Total Parenteral Nutrition (TPN), and had a history of abdominal surgery. Hospital flora generally includes Candida albicans, which is sensitive to azoles. Although azoles were not used, azole-resistant Candida glabrata was an unexpected agent.

While the mortality rate in the first seven days was two (1.45%) in the admitted patients, the 30-day mortality was eight (5.83%). This rate was 19.3% (43/223) in the study of Keven, et al, and the mortality rate was 8.6% (527) in the study of Tanaka, et al.^10,16^ After the Great Marmara earthquake, 5,302 hospitalized patients were involved in a study conducted in 35 reference hospitals, which reported a mortality rate of 4.3% (425).^9^ In this study, while the mortality rate was two (1.45%) people in seven days, the mortality rate was five (3.64%) people at the end of 28 days. Of the patients who died, the cause of death was sepsis due to respiratory tract diseases in three, peptic ulcer perforation in one, and massive pulmonary embolism in one. A high incidence of pneumonia was observed among the elderly in the Hanshin-Awaji earthquake; this was probably due to a sharp deterioration in living conditions, sanitation, and hygiene due to the earthquake, as well as dust inhalation from collapsed buildings.^10^ The causes of death are similar, but the low number of multiple traumas and the absence of severe crush syndrome patients affect the results. The death rates in the study were quite low in the disaster that caused such great destruction. The main reason for the low death rates is the distance to the earthquake zone and the presence of large and fully equipped hospitals close to the earthquake zone. It is concluded that the treatment of patients in critical condition is provided in hospitals close to earthquake areas. Many patients arrived at these regions using their own means and with the help of their relatives. This suggests that the patients are in good condition in many respects.

## Limitations

One of this study’s limitations is that it is single-centered. Furthermore, the distance of the hospital from the disaster zone and the evacuation of large provincial centers closer to the disaster zone resulted in a lower number of patients with serious trauma coming to this hospital, leading to a decrease in total number of applications.

## Conclusions

This research reveals specific data in terms of patient load, patient distribution, infection profile, and death rates in an ED far from the earthquake zone after a major devastation. Factors other than community-acquired factors cause pneumonia and wound infections in earthquake-affected patients, and they also provide data on resistant bacterial growth. Good organization and correct management of all units of the health system will provide appropriate and correct interventions to victims after the disaster.
